# Preclinical Immunogenicity and Efficacy of a Multiple Antigen-Presenting System (MAPS^TM^) SARS-CoV-2 Vaccine

**DOI:** 10.3390/vaccines10071069

**Published:** 2022-07-03

**Authors:** Brian Cieslewicz, Daniel Makrinos, Heidi Burke, Dara Bree, Renuka Haridas, Ian Tonkiss, Yannic Bartsch, Galit Alter, Richard Malley, Gilles Besin

**Affiliations:** 1 Affinivax, Cambridge, MA 02142, USA; brian.cieslewicz@affinivax.com (B.C.); daniel.makrinos@modernatx.com (D.M.); heidi.burke@affinivax.com (H.B.); dara.bree@affinivax.com (D.B.); renuka.haridas@affinivax.com (R.H.); ian.tonkiss@affinivax.com (I.T.); rick.malley@affinivax.com or; 2 Ragon Institute of MGH, MIT, and Harvard, Cambridge, MA 02139, USA; ybartsch@mgh.harvard.edu (Y.B.); galter@mgh.harvard.edu (G.A.); 3 Division of Infectious Diseases, Department of Medicine, Boston Children’s Hospital, Boston, MA 02115, USA

**Keywords:** SARS-CoV-2 vaccine MAPS

## Abstract

Despite the remarkable success of SARS-CoV-2 vaccines, the rise of variants, some of which are more resistant to the effects of vaccination, highlights the potential need for additional COVID-19 vaccines. We used the Multiple Antigen-Presenting System (MAPS) technology, in which proteins are presented on a polysaccharide polymer to induce antibody, Th1, Th17 and CD8+ T cell responses, to engineer a novel vaccine targeting SARS-CoV-2. This vaccine contains a fragment of the spike (S) protein receptor-binding domain (RBD) sequence of the original D614G strain and was used to immunize nonhuman primates (NHP) for assessment of immunological responses and protection against SARS-CoV-2 challenge. The SARS-CoV-2 MAPS vaccine generated robust neutralizing antibodies as well as Th1, Th17 and cytotoxic CD8 T-cell responses in NHPs. Furthermore, MAPS-immunized NHPs had significantly lower viral loads in the nasopharynx and lung compared to control animals. Taken together, these findings support the use of the MAPS platform to make a SARS-CoV-2 vaccine. The nature of the platform also could enable its use for the inclusion of different variants in a single vaccine.

## 1. Introduction

In December 2019, a novel coronavirus was identified following a respiratory disease outbreak in Wuhan, China. This virus, designated Severe Acute Respiratory Syndrome Coronavirus 2 (SARS-CoV-2), causes respiratory disease and other systemic symptoms in humans, defined as coronavirus disease 2019 (COVID-19). Several COVID-19 vaccines have been either authorized or approved, at unprecedented speed, resulting in rapid and high vaccine coverage in several countries. However, despite these impressive achievements, there are increasingly worrisome signs that vaccine-induced immunity may be short-lived, with a rise in breakthrough infections stemming from new variants of SARS-CoV-2 [1]. As a result, it has become doubtful that these first-generation vaccines will provide sufficient control of the virus worldwide.

While several vaccine platforms have been used (Pfizer [2,3], Moderna [4], J&J [5], Novavax [6,7]), the most successful strategies to date have involved mRNA- or DNA-based vaccines. Unlike conventional vaccines that stimulate the immune system through the use of a weakened, damaged, or inactivated version of a pathogen (virus or bacteria), DNA and mRNA vaccines use genetic materials that code for the SARS-CoV-2 spike protein to trigger an immune response. Specifically, DNA vaccines use small DNA molecules (plasmids), while mRNA vaccines use the pathogen’s messenger RNA. Despite some similarities, DNA and mRNA vaccines have several notable differences. Aside from the genetic material used in producing the actual vaccines, they differ in terms of mode of action as well as storage requirements.

DNA vaccines make use of plasmids that carry the gene coding for the SARS-CoV-2 spike protein. Upon entering the human cell, the plasmid should successfully penetrate the cytoplasm and nuclear membrane before it can gain entry to the cell nucleus. Once inside the nucleus, the DNA sequence is converted into messenger RNA (mRNA) which then move back to the cytoplasm, where it is transcribed into protein. Because the particular protein is recognized as a non-self protein by the immune system, the presence of this protein lead to the production of antibodies against the foreign antigen. While DNA vaccines need to enter the nucleus and go all the way back to the cytoplasm to synthesize the necessary viral or bacterial proteins, mRNA vaccines need to reach the cytoplasm, the component of the cell that contains the enzymes necessary for the synthesis of the bacterial or viral proteins.

Despite the required specific delivery into the nucleus, DNA vaccines are significantly more temperature-stable compared to mRNA vaccines. Between the two, plasmid DNA vaccines are more stable and are easier to store and transport, while mRNA vaccines have stringent storage and transportation requirements, which significantly hamper the distribution process to poorer nations. Conversely, because of its direct delivery to the nucleus, the mRNA generates a faster and massive expression of the foreign antigen leading to robust antibody response quicker.

The SARS-CoV-2 DNA- and mRNA-based vaccines generate robust, neutralizing antibodies directed against the S protein of SARS-CoV-2. Questions about durability and breadth of immune responses remain, particularly given growing evidence to support a role of T cells in protection [8].

The multiple antigen-presenting system (MAPS) enables the creation of a macromolecular complex that mimics the properties of attenuated cells vaccines by integrating various antigen components, including polysaccharides and proteins, in the same construct and that induce multipronged immune responses, including antibody, Th1, and Th17 responses. Using antigens from various pathogens (*Streptococcus pneumoniae*, *Salmonella typhi*, and *Mycobacterium tuberculosis*), the versatility of the MAPS system and its feasibility for the design of unique defined-structure subunit vaccines to confer comprehensive protection via multiple immune mechanisms has been demonstrated [9]. In addition, this work demonstrated that T cell generated by MAPS is correlated with secretion of proinflammatory cytokines environment (TNF-α, IL-12, IL-23, and IL-1β) and depends on TLR2 activation, in a dose-dependent manner [9].

AFX3772, a MAPS vaccine consisting of 24 pneumococcal polysaccharides and a fusion protein comprising two pneumococcal proteins and rhizavidin, has been shown to be safe and immunogenic in healthy adult and older adults [10].

**Figure 1:**
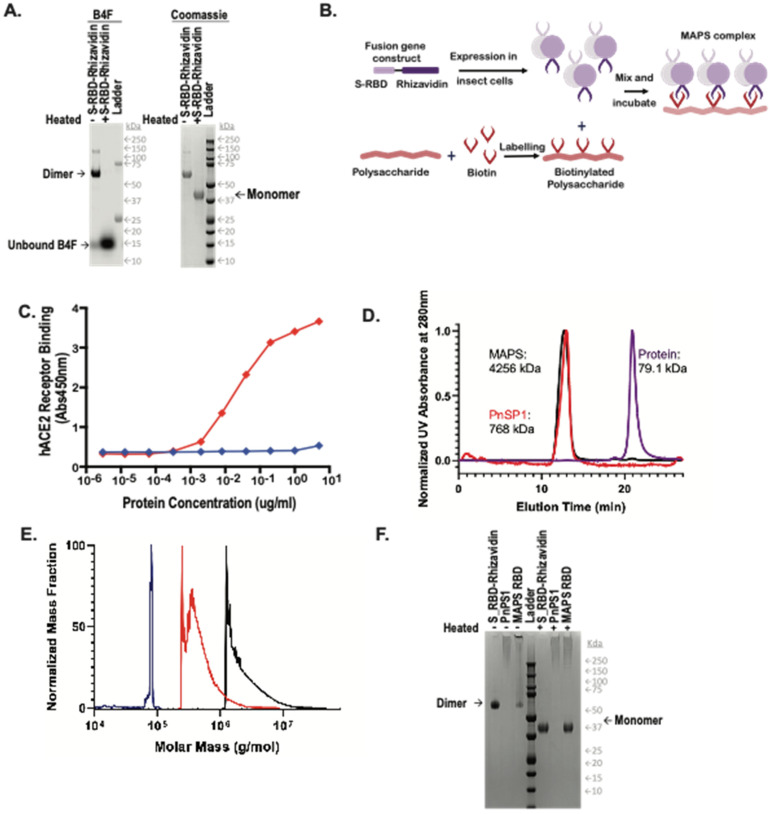
Generation of MAPS complexes. (**A**) Expression and purification via nickel chromatography of the RBD-Rhizavidin fusion protein. (Left) fluorescein detection of biotin-4-fluorescein (B4F), (right) Coomassie stained SDS-PAGE. A 1 μg amount of each sample was loaded with 1× reducing Laemmli buffer, (−) indicates samples were not heated and (+) indicates samples were heated for 10 min at 100 °C. (**B**) Schematic diagram of MAPS technology. (**C**) Binding of S-RBD-Rhizavidin to the human ACE2 receptor. (**D**) HPLC-SEC Chromatograms of S_RBD-Rhizavidin shown in purple, pneumococcal polysaccharide type 1 (PnSP1) shown in red, and MAPS complex shown in black, absorbance at 280 nm. (**E**) Mass distribution of samples via SEC-MALS, S_RBD-Rhizavidin shown in purple, PnSP1 shown in red, and MAPS complex shown in black. (**F**) Coomassie stained SDS-PAGE of RBD-Rhizavidin fusion protein, PnSP1, and MAPS complexes. ~1 μg of each sample was loaded with 1× reducing Laemmli buffer, samples on the left side of the ladder were not heated (−) and samples to the right of the ladder were heated for 10 min at 100 °C (+).

In the present study, we make use of the Multiple Antigen-Presenting System (MAPS), which can induce robust antibody and T-cell responses. MAPS combines pathogen-specific proteins fused to rhizavidin (an avidin-like protein) with biotinylated polysaccharides, resulting in the formation of a complex that can elicit antibodies to all components, and T-cell responses to proteins [11]. Here, a RBD fragment of the original SARS-CoV-2 strain (D614G) was genetically fused to rhizavidin fragment and purified from insect Sf9 cells. This fusion protein was then combined with a biotinylated polysaccharide (type 1 from Streptococcus pneumoniae) to form a MAPS complex [12,13] (see Figure 1B). This SARS-CoV-2 MAPS vaccine was then tested in rabbits and in non-human primates (NHP) for evaluation of immunogenicity and protection against viral challenge.

## 2. Materials and Methods

### 2.1. Construction, Expression, and Purification of S-RBD-Rhizavidin Fusion Antigens

The fusion protein S-RBD-Rhizavidin was obtained by fusing the S-RBD sequence (fragment corresponding to AA 331-524) to the gene sequence encoding the rhizavidin (Rhizavidin) fragment as previously described [14]. The fusion construct obtained by synthetic synthesis was cloned into the pFastBac1 plasmid pre-digested with the EcoRI and HindIII restriction enzymes using cloning kit. DH10Bac strain was used for the recombinant bacmid (rbacmid) generation. The positive bacmid containing S-RBD-Rhizavidin sequence gene was confirmed by PCR. Sf9 cells were grown in Sf-900 II SFM Expression Medium (Life Technologies, Carlsbad, CA, USA). The cells were maintained in Erlenmeyer Flasks at 27 °C in an orbital shaker. One day before transfection, the cells were seeded at an appropriate density in 6 wells. On the day of transfection, DNA and transfection Reagent (Promega, Madison, WI, USA) were mixed at an optimal ratio and then added into the plate with cells ready for transfection. Cells were incubated in Sf-900 II SFM for 5–7 days at 27 °C before harvest.

His-tagged recombinant proteins were purified using nickel-nitrilotriacetic acid (NTA) affinity chromatography. To improve purity, the eluents of the affinity column were then subjected to SEC on a Superdex 200 column. The peak fractions containing the fusion proteins were collected, evaluated by SDS/PAGE, and then flash-frozen in liquid nitrogen for future use. Protein concentration was measured using a bicinchoninic acid (BCA) protein assay kit (Pierce).

### 2.2. Manufacture of SARS-CoV-2 MAPS

Each MAPS complex was assembled by incubation of biotinylated polysaccharide with fusion antigens at 4 °C overnight. The assembled complex was isolated by Size Exclusion Chromatography (SEC), using Tris-buffered saline (20 mM Tris-HCl pH 8.0, 150 mM NaCl) as an eluant. The fractions containing MAPS complex were pooled. The protein concentration in a MAPS complex was measured using a bicinchoninic acid (BCA) protein assay kit (Pierce), and the polysaccharide concentration was determined by uronic acid assay. Uronic acid assay is a colorimetric assay used to determine concentration of polysaccharide via reaction with m-hydroxydiphenyl. Polysaccharide is heated in the presence of sulfuric acid to hydrolyze the polysaccharide containing uronic acid. The uronic acid then reacts with m-hydroxydiphenyl under acidic conditions to produce a pink solution that absorbs at 520 nm. Sodium tetraborate is included in the reaction to enhance the intensity of the color change. Concentration of the sample is then defined by comparison of sample absorbance to a serial dilution of D-galacturonic acid at a known concentration.

### 2.3. Evaluation of SARS-CoV-2 MAPS Vaccine by Analytical HPLC-SEC-MALS

Samples were analyzed via HPLC-SEC. Approximately 20 micrograms of each sample were injected onto an AdvanceBio SEC 1.9 μm column (Agilent Technologies, Santa Clara, CA, USA) on Waters Acquity HPLC. Samples were flowed over the column at 0.4 mL/min in 20 mM HEPES, 100 mM sodium chloride at 25 °C maintaining pre-column pressure of approximately 1500 PSI. In line detectors measured UV absorbance at 280 nm following the column. Absorbances were normalized to the maximum absorbance within each run and plotted against one another. Following separation via HPLC-SEC sample was flowed through Wyatt Dawn, an 18 angle Multi-Angle Light Scattering (MALS) detector with a source laser emitting at 658 nm, and Wyatt Optilab differential refractometer with a laser emitting also at 658 nm. Data from these were analyzed via Astra 8.0 software, in which samples were gated at full width at half maximum while using a first order Zimm fit to generate molecular weight data.

### 2.4. Evaluation of S-RBD-Rhizavidin Protein and SARS-CoV-2 MAPS Vaccine by SDS-PAGE

Samples were diluted with water and reducing Laemmli buffer. Additionally, biotin-4-fluorescein (B4F) (Invitrogen, Waltham, MA, USA) was added to the protein purification samples at a final concentration of 2 mg/mL. Samples were then either incubated at room temperature (RT) or heated at 100 °C for ten minutes. One microgram of each sample was loaded on a 4–12% Bis-Tris Protein gel (Invitrogen, Waltham, MA, USA) and then ran for 40 min at 200 volts. The gel was then stained with InstantBlue Coomassie Protein Stain (Abcam, Cambridge, UK) for 15 min and rinsed with water. The gel was exposed for 0.5 s on the Bio-Rad ChemiDocMP imager.

### 2.5. Determination of S-RBD-Rhizavidin Binding to hACE2 Receptor

Plates were precoated with 5 µg/mL of hACE2 receptor overnight and washed with phosphate-buffered saline with 0.05% Tween (PBS-T) buffer and blocked with TBS Startblock blocking buffer (Thermo Fisher Scientific, Waltham, MA, USA). Histidine-tagged S-RBD-Rhizavidin was threefold serially diluted and added to coated wells for 2 h at RT. Following washing, 100 µL of anti His-HRP (Bio-Rad, Hercules, CA, USA) was added to the plate. Plates were incubated for 1 h at RT, while SureBlue TMB Microwell Peroxidase Substrate (VWR, Radnor, PA, USA) equilibrated to RT. After a final wash, 100 µL TMB substrate was added to wells and development was stopped with 100 µL of 1N hydrochloric acid after 10 min at RT. The ELISA plates were read at an absorbance of 450 nm on a SpectraMax i3x Plate Reader using Softmax Pro 7.0.

### 2.6. Rabbit Immunization

All rabbit immunizations were performed at Cocalico Biologics (Stevens, PA, USA). Female New Zealand White rabbits (n = 3/group) were injected intramuscularly (IM) with either 50 µg of SARS-CoV-2 MAPS vaccine or saline vehicle (0.5 mL). Booster injections were given at Day 21 and Day 42 post-first injection. Sera were obtained before each immunization and at a terminal bleed on Day 56 for measurement of antibodies. Observations for morbidity, mortality, clinical signs, body temperature, food and water consumption were conducted on a regular basis for all animals.

### 2.7. Non-Human Primate Immunization and Challenge

The study was performed by BIOQUAL, Inc. (Rockville, MD, USA). The study was conducted in accordance with the Study Protocol and BIOQUAL Standard Operating Procedures (SOPs) when applicable, and in accordance with the animal welfare requirements and accreditations stated below. The study was performed in compliance with the following regulations or guidelines: Housing and handling of the animals were performed in accordance with the standards of the AAALAC International’s reference resource: the 8th edition of the Guide for the Care and Use of Laboratory animals, Animal Welfare Act as amended, and the 2015 reprint of the Public Health Service (PHS) Policy on Human Care and Use of Laboratory Animals. Handling of samples and animals occurred in compliance with the Biosafety in Microbiological and Biomedical Laboratories (BMBL), 5th edition (Centers for Disease Control). This study was performed under an IACUC approved protocol.

A total of 12 adult male, cynomolgus macaques (Mauritian origin, Macaca fascicularis), about 3–4 kg at time of delivery, were purchased from PreLabs (Lehigh Acres, FL, USA). In vivo procedures were performed according to the approved animal protocols at Bioqual Inc. All components of the study were conducted at Bioqual Inc. (Baltimore, MD, USA) except for the intracellular cytokine staining, which was conducted at Affinivax (Cambridge, MA, USA).

The animals (n = 6/group) were injected subcutaneously (SQ) with either 100 µg of SARS-CoV-2 MAPS vaccine or saline vehicle (1 mL). A booster injection was given at Day 21 post-first injection. Sera were collected prior to and following each immunization to evaluate induction of antibody responses and cellular immunity to the vaccine antigen. Blood was collected to isolate PBMCs at Day 42 from animals immediately prior to challenge with 10^5^ PFU/2 mL of SARS-CoV-2 USA-WA1/2020 isolate via the combination intranasal/intratracheal (IN/IT) route. Nasal swabs and BAL were collected throughout the post challenge phase to monitor viral load over time. Animals were monitored for symptoms through post-challenge from Days 42–56 followed by euthanasia for necropsy and tissue collection.

### 2.8. Isolation of Peripheral Blood Mononuclear Cells (PBMCs)

CPT tubes were spun as follows for isolation of PBMCs: 2700 rpm for 30 min followed by 3 spins/washes at 1500, 1200, and 1000 rpm each for 10 min. The live vs. dead PBMCs were enumerated using a Nexcelom Cellometer K2 (SOP# BV-031). The PBMCs were then resuspended in FBS with 10% DMSO using Mr. Frosty^®^ (Thermo Scientific, Waltham, MA, USA) or similar freezing boxes. The boxes were placed immediately into a −80 °C freezer for 24 h and then transferred for storage in a liquid nitrogen tank. The number of cells per vial is typically 5–10 million and viability is normally >90%.

### 2.9. SARS-CoV-2 S, S Subunit, RBD, and N IgG ELISA

Sandwich ELISA was performed to quantify serum IgG levels. Nunc-Immuno MaxiSorp 96-well plates were coated with 4 µg/mL of SARS-CoV-2 S-RBD, his-tag (Wild-Type, SPD-C52H3, Acro biosystems, Newark, DE, USA), SARS-CoV-2 S-RBD (N501Y), his-tag (UK Mutant, SPD-C52Hn, Acro biosystems, DE), S-P2 Spike, his-tag (Trimer, UWashington, 35962), RBD Soluble, his-tag (UWashington, 35961), and SARS-CoV-1 (S1 Subunit) S-RBD (40150-V08B1, Sino Biological, Chester Brook, PA, USA) and left overnight at RT. Similarly, AffiniPure F(ab’)2 Fragment-specific goat anti-human IgG (Jackson Laboratory, Bar Harbor, ME, USA) was coated for standards. IgG ELISA plates were washed (BioTek 405 TS) in 1× DPBS-T (0.05% Tween-20) and blocked with 1% bovine serum albumin (BSA) (Millipore Sigma, St. Louis, MO, USA) for 1 h at RT. After blocking, plates were washed and 100 µL of diluted sera/purified human IgG (MP Biomedicals, Irvine, CA, USA) were added to the antigen-coated plate and incubated for 1 h at RT. Following washing, a Goat Anti-Human (H + L)-HRP (Bio-Rad, Hercules, CA, USA) was diluted to 1:20,000 in 1× DPBS-T and added 100 µL/well to the plate. Plates were incubated for 1 h at RT, while SureBlue TMB Microwell Peroxidase Substrate (VWR, Radnor, PA, USA) equilibrated to RT. After a final wash, 100 µL TMB substrate was added to wells and development was stopped with 100 µL of 1N hydrochloric acid after 10 min at RT. The ELISA plates were read at an absorbance of 450 nm on a SpectraMax i3x Plate Reader using Softmax Pro 7.0.

### 2.10. Plaque Reduction Neutralization Test (PRNT)

SARS-CoV-2 neutralization was assessed as previously described [14]. In brief, a pre-titrated dose of virus was incubated with 8 serial 5-fold dilutions of serum samples in duplicate in a total volume of 150 μL for 1 h at 37 °C in 96-well flat-bottom poly-L-lysine-coated Biocoat plates (Corning, New York, NY, USA). Cells were suspended using TrypLE Select Enzyme solution (Thermo Fisher Scientific, Waltham, MA, USA) and immediately added to all wells (10,000 cells in 100 μL of growth medium per well). One set of 8 control wells received cells + virus (virus control) and another set of 8 wells received cells only (background control) in a volume of 100 μL. After 66–72 h of incubation, medium was removed by gentle aspiration and 30 μL of Promega 1× lysis buffer was added to all wells. After a 10-min incubation at RT, 100 μL of Bright-Glo luciferase reagent was added to all wells. After 1–2 min, 110 μL of each cell lysate was transferred to a black/white plate (Perkin-Elmer). Luminescence was measured using a PerkinElmer Life Sciences, Model Victor2 luminometer. Neutralization titers are the serum dilution at which relative luminescence units (RLU) were reduced by 50% (ID_50_) compared to virus control wells after subtraction of background RLUs. Serum samples were heat-inactivated for 30 min at 56 °C prior to the assay.

### 2.11. Antibody-Dependent Complement Deposition

Antibody-dependent complement deposition (ADCD) was assessed as described previously [15]. In brief, biotinylated antigen was coupled to fluorescent NeutrAvidin beads (Thermo Fisher Scientific, Waltham, MA, USA). Plasma antibodies were diluted 1:10 in 0.1% BSA and incubated with the coupled antigen beads for 2 h at 37 °C. Beads were washed and incubated with complement factors from guinea pig for 20 min at 37 °C. The complement reaction was then stopped by washing with 15 mM EDTA in PBS. C3 deposition on the beads was detected with a 1:100 diluted FITC-conjugated anti-guinea pig C3 polyclonal antibody (MP Biomedicals), and relative C3 deposition was analyzed by flow cytometry.

### 2.12. Antibody-Dependent Neutrophil Phagocytosis

Antibody-dependent neutrophil phagocytosis (ADNP) was assessed as described previously [16]. Briefly, primary human neutrophils were obtained from ACK lysed blood of healthy donors. Biotinylated antigens were incubated with NeutrAvidin beads and ICs formed by incubation with 1:100 diluted plasma for 2 h at 37 °C in 96-well plates (Greiner Bio-One, Kremsmünster, Austria). Isolated neutrophils were added afterwards and incubated for 1 h at 37 °C. Neutrophils were surface stained against CD66b (1:50, Biolegend, San Diego, CA, USA, clone: G10F5), fixed with 4% paraformaldehyde and analyzed on an LSRII (BD) flow cytometer. Phagocytosis score was calculated as the product of frequency beads positive CD66b neutrophils and bead fluorescent intensity using FlowJo 10.8.

### 2.13. Antibody-Dependent Cellular Phagocytosis

Antibody-dependent cellular phagocytosis (ADCP) was assessed as described previously [17]. In brief, THP-1 monocyte phagocytosis was performed as previously described. Briefly, biotinylated antigens were conjugated to NeutrAvidin beads and incubated with 1:100 diluted plasma samples. THP-1 monocytes (0.25 million cells per well) were added to the ICs and incubated for 16 h at 37 °C, fixed with 4% paraformaldehyde and analyzed by flow cytometry.

### 2.14. IgG Subclass, Isotype and FcR-Binding Luminex Profiling

IgG subclass and FcR profiling was conducted as previously described [18,19]. Briefly, antigens were carboxyl coupled to magnetic Luminex microplex carboxylated beads (Luminex Corporation, Austin, TX, USA) using NHS-ester linkages with Sulfo-NHS and EDC (Thermo Fisher, Waltham, MA, USA), and then incubated with serum for 2 h at room temperature. Subclass (IgG1 or IgG3) titer were first probed with a mouse rhesus-subclass IgG1 or IgG3 specific secondary antibody (NHP Reagent Resource), respectively; mouse IgG was then detected with a PE-conjugated anti-mouse antibody (Thermo-Fisher). FcR binding was quantified by incubating immune complexes with biotinylated FcRs (FcγR2A-1, FcγR2A-2, FcγR3A, Duke Protein Production Facility) conjugated to Steptavidin-PE (Agilent, Santa Clara, CA). Flow cytometry was performed with an IQue (Intellicyt, Albuquerque, NM, USA), and analysis was performed on IntelliCyt ForeCyt (v8.1).

### 2.15. Subgenomic mRNA Assay

Replicating virus load by qRT-PCR targeting the subgenomic envelope (E) gene RNA in 250 mL aliquot of nasopharyngeal swabs, nasal washes, and BAL aspirates. The forward and reverse primers, probe used here were:

SUBGEN-FORWARD: 5′-CGATCTCTTGTAGATCTGTTCTC-3′

E_Sarbeco_R2 Reverse rimer: 5′-ATATTGCAGCAGTACGCACACA-3′

Probe (Thermo): FAM-MGB: 5′-ACACTAGCCATCCTTACTGCGCTTCG-3′

### 2.16. Infectious Viral Load (TCID_50_) Assay

Vero E6 cells (ATCC no. CRL-1586) were plated at 25,000 cells/well in 200 μL of DMEM + 10% FBS + Gentamicin and the cultures are incubated at 37 °C, 5.0% CO_2_. Cells were 80–100% confluent the following day. Medium was aspirated and replaced with 180 μL of DMEM + 2% FBS + gentamicin. Twenty (20) μL of sample was added to top row in quadruplicate and mixed using a P200 pipettor 5 times. Using the pipettor, 20 μL was transferred to the next row, and repeated down the plate (columns A–H) representing 10-fold dilutions. Positive (virus stock of known infectious titer in the assay) and negative (medium only) control wells were included in each assay set-up. The plates were incubated at 37 °C, 5.0% CO_2_ for 4 days. The cell monolayers were visually inspected for CPE. The TCID_50_ value was calculated using the Read-Muench formula (log_10_ 50% end point dilution = log_10_ of dilution showing a mortality next above 50%-(difference of logarithms × logarithm of dilution factor).

## 3. Results

### 3.1. Manufacture of SARS-CoV-2 MAPS

Expression of S-RBD-Rhizavidin fusion protein in eukaryotic cells: The fusion protein S-RBD-Rhizavidin was obtained by fusing the S-RBD sequences (fragment corresponding to AA 331-524) to the gene sequence encoding the rhizavidin (fragment corresponding to AA 45-179) and cloned into pFastBac1 plasmid; the baculovirus expression system was used to express the fusion construct in SF900II cells. This His-tagged recombinant protein was then purified using nickel-nitrilotriacetic acid (NTA) affinity chromatography and was analyzed using SDS-PAGE (Figure 1A). The rhizavidin dimer is very stable and remains intact in reducing SDS buffer, maintaining its ability to bind biotin-4-fluorescein (B4F), and dissociates only after heating to 100 °C in reducing SDS [12].

Human Angiotensin Converting Enzyme 2 (hACE2) receptor binding assay: To demonstrate the preservation of the relevant S-RBD protein epitopes, we performed an hACE2 receptor binding assay. The data demonstrated strong binding of the S-RBD-Rhizavidin fusion protein to the hACE2 receptor in a concentration-dependent matter, thus indicating that the RBD protein in the fusion remained functional (Figure 1C).

MAPS formation: MAPS complex was assembled by incubation of biotinylated polysaccharide with the fusion protein at 4 °C overnight then purified via preparative size exclusion chromatography. Purified MAPS and components were examined by HPLC-SEC (Figure 1D), larger molecules of MAPS and polysaccharide eluted at the void volume of t = ±12 min, while unlinked protein antigens are retained longer and elute later from the column (t = ±21 min). Molecular weights of the complexes were compared using multi-angle light scattering (MALS), showing that MAPS complexes had a weight average molecular weight of 4256 kDa, polysaccharide 768 kDa, and S-RBD-Rhizavidin dimer of 79.1 kDa in figure (Figure 1E).

As shown previously [12], the association between polysaccharide and protein antigens in MAPS is very stable, resistant to treatment with reducing SDS buffer, and undergoes dissociation only upon heating to 100 °C in SDS, allowing for estimation of assembly efficiency by comparing the SDS-PAGE antigen profiles of unheated and heated samples [12]. The incorporation of S-RBD-Rhizavidin fusion protein into MAPS complexes was confirmed by SDS-PAGE of the purified MAPS complex (MAPS non-heated and heated sample lanes) (Figure 1F). In the heated samples there is a band corresponding to the monomer of the target antigen whereas in the non-heated MAPS sample, the fusion protein does not migrate, as it is retained with the affinity-linked polysaccharide in the well.

### 3.2. Immunogenicity of the SARS-CoV-2 MAPS in Rabbits

**Figure 2:**
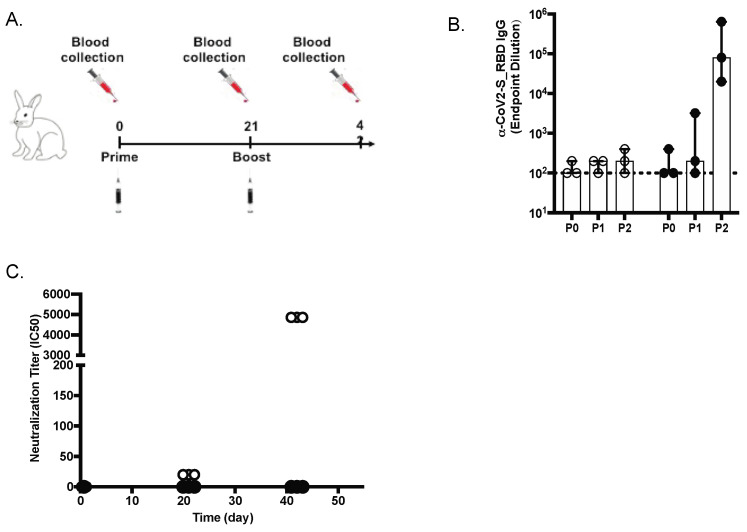
Anti-spike (S) IgG and neutralizing antibodies in rabbits. (**A**) Design of the rabbit in vivo study (**B**) Anti-S IgG levels (ELISA; OD 450 nm) at days 21 and 42 are shown for placebo (white diamonds) and vaccinated animals (black diamonds). (**C**) SARS-CoV-2 virus 50% serum neutralizing titers (VNT50) of sera at days 0, 21, and 42 are shown for placebo and vaccinated animals.

New Zealand rabbits (n = 3) were immunized with two subcutaneous inoculations of SARS-CoV-2 S-RBD MAPS vaccine at Day 0 and 21 (Figure 2A). The antibody responses against SARS-CoV-2 S protein were measured on Day 0 (baseline before injection, P0), Day 21 (representing the response after the first immunization, P1), and again on Day 42 (representing the response after the second immunization, P2) by ELISA and virus neutralization assay (VNT). We noted an increase in serum concentration of antibodies to S and neutralizing antibody activity, especially after the second dose (Figure 2B,C).

### 3.3. Immunogenicity of the SARS-CoV-2 MAPS Vaccine in Cynomolgus Macaques

**Figure 3:**
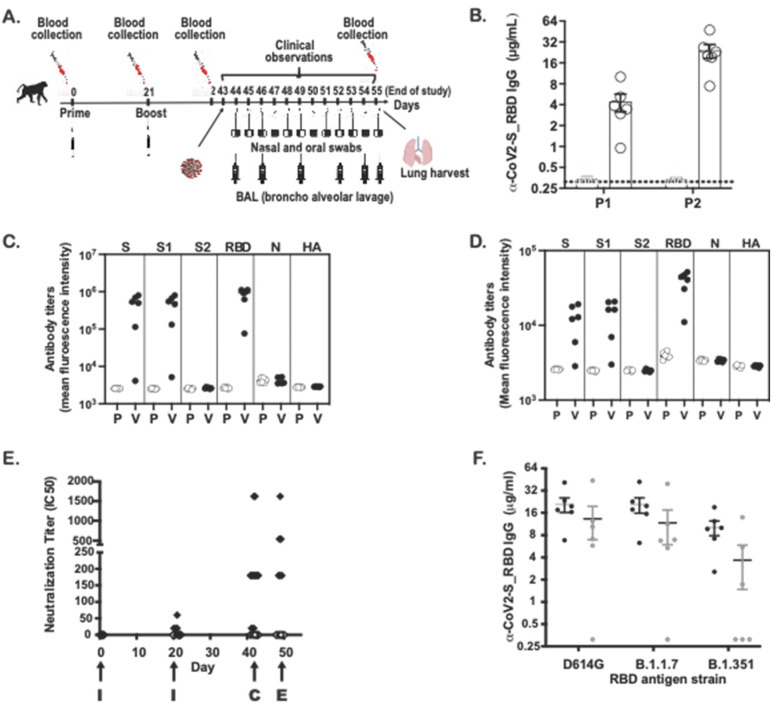
Anti-spike (S) IgG and neutralizing antibodies in non-human primates. (**A**) Design of the NHP in vivo study (**B**) Anti-S IgG levels (ELISA; OD 450 nm) at days 21 (P1) and 42 (P2) are shown for placebo (P) and vaccinated (V) NHP groups. (**C**) IgG1 levels against S, S1, S2, RBD, N and HA flu at day 42 are shown for placebo and vaccinated NHP groups. (**D**) IgG3 levels against S, S1, S2, RBD, N from SARS-CoV-2 virus and HA flu virus at day 42 are shown for placebo and vaccinated NHP groups. (**E**) I SARS-CoV-2 virus 50% serum neutralizing titers (VNT50) of sera at days 0, 21, 42 and 49 are shown for placebo (white diamonds) and vaccinated NHP groups (black diamonds). I = immunization, C = challenge, E = euthanasia. (**F**) IgG levels against S-RBD protein from D614G or B1.1.7 or B1.351 are shown from sera of vaccinated NHP groups (day 42 serum) (black circles) or from sera coming from seroconverted patients (n = 6) (white circles).

Adult Cynomolgus macaques (n = 6, males, 3.3–5.0 years-old) were immunized with two subcutaneous inoculations of SARS-CoV-2 MAPS vaccine at Day 0 and 21 (Figure 3A). An age-matched control group (n = 6, males, 3.3–5.0 years-old) received an injection of saline at Day 0 and 21. SARS-CoV-2 S–specific antibody responses were measured 21 days post first injection, before the second injection (P1) and again 21 days post second injection (P2) by ELISA and virus neutralization assay (VNT). Figure 3B shows the increase in antibodies to S after the first and second immunizations. The concentration of RBD-specific subclass IgG was measured by Luminex-based assay: IgG1 (Figure 3C). and IgG3 (Figure 3D) antibodies directed against S and S1 domain were detected, but this was not the case for the S2 domain, which is not included in the vaccine. The specificity of the measured response was further confirmed by the absence of ELISA signal to SARS-CoV-2 nucleoprotein (N) and the influenza HA protein. Similar to the IgG responses in ELISA, we detected neutralizing antibody responses at Day 21 (Figure 3E), which increased even further after the second immunization (Figure 3E).

To evaluate for the presence of cross-reactive antibodies against variant strains, we compared the concentration of antibodies directed against the RBD sequence of the D614G, alpha (B1.1.7), or gamma (B1.351) variant in NHP sera following immunization with SARS-CoV-2 MAPS. In the same experiment, sera from patients who recovered from COVID-19 were also included, to evaluate the presence of cross-reactive antibodies in human (Figure 3F). The concentration of total IgG directed against S-RBD was highest against the RBD derived from the D614G strain compared to that directed against either of the two variants. We observed similar results from immunized animals and from human convalescent sera.

### 3.4. Evaluation of the Effector Function of SARS-CoV-2 MAPS-Induced Antibodies

Opsonophagocytic and cytotoxic function depend on the ability of antibodies to interact with Fc-receptors found on immune cells [20]. In humans and nonhuman primates, four low-affinity Fc receptors (FcγR2a, FcγR2b, FcγR3a, and FcγR3b) drive IgG-mediated activation [21].

**Figure 4:**
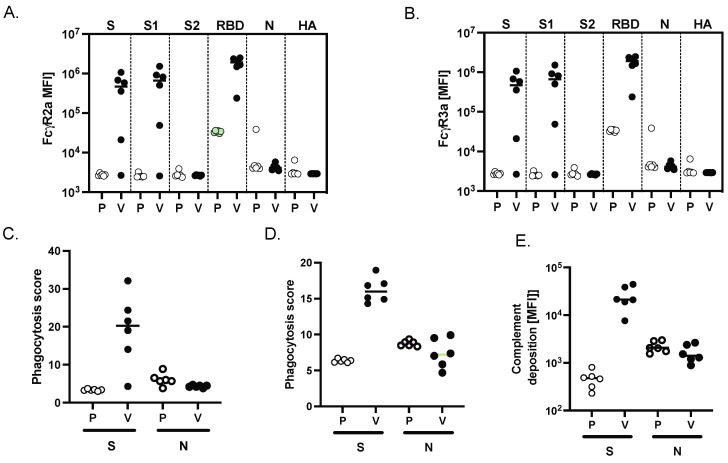
MAPS vaccine induces FcγR binding and phagocytic activity in NHP. Binding of SARS-CoV-2 specific antibodies to FcγR2a (**A**) and FcγR3a (**B**) was determined by Luminex in sera from placebo (P) and vaccinated NHP (V) groups. (**C**) Using the S or the N SARS-CoV-2 protein, the ability of SARS-409 CoV-2 S specific antibody Fc to induce antibody-dependent-complement-deposition (ADCD) (**C**), neutrophil-phagocytosis (ADNP) (**D**), or cellular-THP1 monocyte-phagocytosis (ADCP) (**E**) was analyzed for placebo and vaccinated NHP groups.

To evaluate the Fc effector function of antibodies generated following two immunizations with SARS-CoV-2 MAPS, we used a systems serology approach published previously [22] to evaluate antibody-dependent cellular phagocytosis (ADCP), antibody-dependent neutrophil phagocytosis (ADNP), and antibody-dependent complement deposition (ADCD). When compared to sera from NHPs that had received saline alone, sera from NHPs immunized with SARS-CoV-2 MAPS bound more to FcγR2a (Figure 4A) and FcγR2b (Figure 4B) in the presence of S, S1 or S-RBD protein. No increase in binding was observed in the presence of control proteins S2, or N SARS-CoV-2, or HA.

Finally, to explore the functional impact of vaccine-induced antibody Fc profiles, we examined cellular monocyte phagocytosis (ADCP, Figure 4C), neutrophil phagocytosis (ADNP, Figure 4D), and complement deposition (ADCD, Figure 4E). All these effector functions were observed in sera obtained from SARS-CoV-2-immunized NHP in the presence of the S protein; as expected, none of these activities was seen in the presence of the N protein.

### 3.5. SARS-CoV-2 MAPS Vaccine Drives a Potent Cellular Immune Response in Cynomolgus Macaques

**Figure 5:**
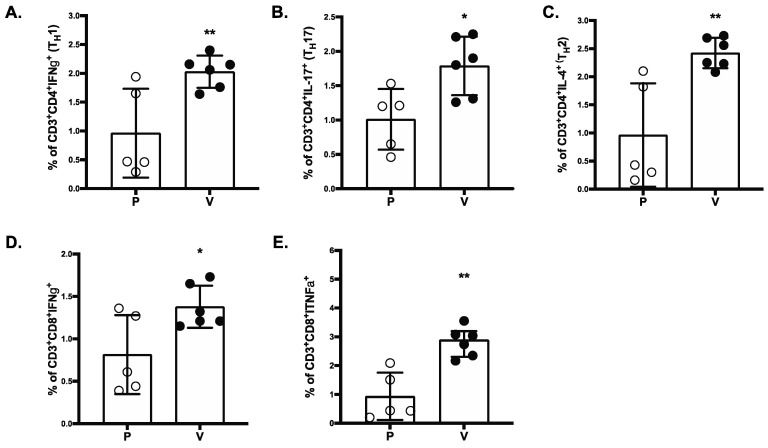
SARS-COV-2 MAPS RBD vaccine induces CD4+ and CD8+ T-cell responses. Splenocytes of placebo (P) or vaccinated (V) NHP groups were re-stimulated ex vivo with full-length S peptide mix or buffer. CD4^+^ Th1 cells (**A**), Th2 cells (**B**), Th17 (**C**), IFNγ^+^ CD8^+^ (**D**), or TNFα^+^ CD8^+^ (**E**) T-cell specific cytokine release by splenocytes collected from NHP 14 days after second immunization with SARS-CoV-2 MAPS RBD vaccine or saline (placebo) were determined by flow cytometry after ex vivo restimulation. S-peptide specific responses are corrected for background (no peptide). * *p* ≤ 0.05, ** *p* ≤ 0.01.

S-RBD-specific T-cell responses were analyzed in vaccinated and placebo NHP groups by intracellular cytokine staining (ICS). The SARSCoV-2 MAPS vaccine generated significantly increased S-RBD-specific IFN-γ-producing T-cell responses, including higher frequencies of CD4+ Th1 cells (Figure 5A), CD8+ T cells (Figure 5D), and TNFα-producing CD8+ T-cells (Figure 5E). Both Th1 and Th17 cell frequency (Figure 5C) were also increased in vaccinated NHPs compared to the placebo group. Finally, significantly greater numbers of IL-4 producing CD4+ T-cells (Figure 5B) were detected in SARS-CoV-2 MAPS vaccinated NHPs compared to placebo vaccinated NHPs.

### 3.6. Protective Efficacy against Upper and Lower-Airway SARS-CoV-2 Infection

**Figure 6:**
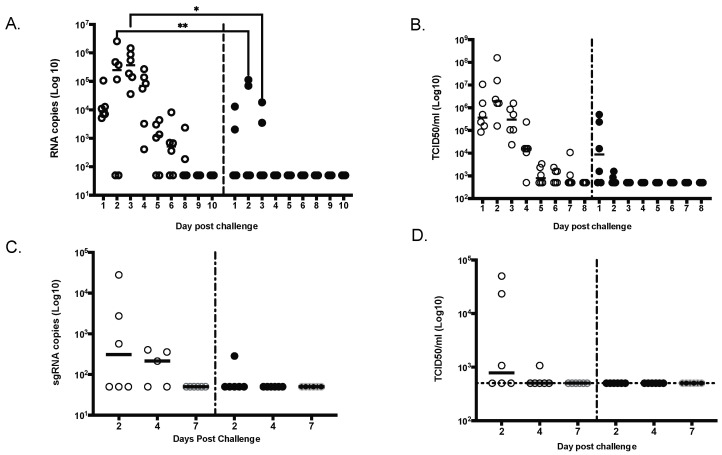
MAPS-immunized NHPs had significantly lower viral load compared to control animals. Viral shedding was measured daily for 9 days in nasal swabs by either qRT-PCR (**A**) or TCID_50_ (**B**) or in the lung (bronchoalveolar lavage) at day 2, 4 and 7 by either sgRNA RT-PCR (**C**) or TCID_50_ (**D**). * *p* ≤ 0.05, ** *p* ≤ 0.01.

All animals were challenged by combined intratracheal and intranasal routes as previously described [23], with a total dose of 10^5^ PFU of the D614G strain of SARS-CoV-2 administered 4 weeks after the second vaccination. Due to the importance of nasal epithelial and pulmonary cells in SARS-CoV-2 infection [24,25,26], both nasal washes and bronchoalveolar lavage (BAL) samples were tested for SARS-CoV-2 RNA by reverse-transcription quantitative polymerase chain reaction (RT-qPCR) or Median Tissue Culture Infectious Dose (TCID_50_) assays (Figure 6). In NHPs that received saline alone, viral RNA was detected in nasal swabs obtained from Day 1 to Day 8 after challenge, both by RT-qPCR (Figure 6A) and TCID_50_ (Figure 6B). In vaccinated NHPs, viral RNA was detected in 2/6 nasal swab by RT-qPCR; by Day 4, all nasal swabs were negative (Figure 6A). By TCID_50_, 4/6 NHP were positive on Day 1 but quickly reverted to negative (Figure 5B). From BAL samples, viral RNA was detected in 3 of 6 unimmunized NHPs on Days 2 and 4 after challenge (Figure 6C,D), whereas viral RNA was detected in the BAL of only one of six immunized NHP at Day 2, and no viral RNA was detected at later timepoints.

Of interest, we observed an increase of the antibody response after the challenge in the vaccinated NHPs (including one animal that had no detectable neutralization antibody after dose 2 and prior to challenge) whereas no neutralizing antibody was detected in the unimmunized NHPs (Figure 3E), suggesting a boosting effect following exposure to the virus.

## 4. Discussion

In response to the COVID-19 pandemic, the scientific and biopharmaceutical community has rallied with unprecedented speed to develop effective vaccines to combat this novel pathogen. Many vaccine platforms have been utilized to develop a COVID-19 vaccine quickly, with several already approved for human use barely within a year of the emergence of the virus. Despite this remarkable success, the rise of SARS-CoV-2 variants, some of which are more resistant to the effects of vaccination, highlights the potential need for additional COVID vaccines. In comparison to DNA- or RNA-based vaccines, protein-based vaccines may be associated with fewer side effects, but are not yet in widespread use for COVID-19, even though late-stage clinical-trial data look promising. In this manuscript, we describe the use of the MAPS vaccine platform, which relies on an affinity-based association between polysaccharides and proteins to elicit immune response against bacterial and viral targets. The findings reported here demonstrate that two doses of this prototype SARS-CoV-2 MAPS in an NHP and in rabbit model induces a robust neutralizing antibody responses. Importantly, the neutralizing antibody responses observed before challenge in vaccinated NHP were comparable to those measured in prior studies using similar assays following 100 μg of the mRNA-based vaccines, mRNA1273 [27] and BNT162b [2], and higher than those measured in convalescent humans.

We also show that immunization with MAPS generates both CD4+ and CD8+ T cells directed against the SARS-CoV-2 RBD. The importance of T cells in disease severity and duration and driving recovery from SARS-CoV-2 infection has been shown in animal and human studies. Notably studies in non-human primates demonstrated that SARS-CoV-2-specific T cells offer a substantial level of protection from SARS-CoV-2 infection and improved outcome [28]. Similarly, T-cell-mediated protection from coronaviruses, including SARS-CoV-2, in mice has been reported [29,30]. Clinically early induction of interferon (IFN)-γ-secreting SARS-CoV-2-specific T cells was associated with milder disease in COVID-19 patients [31]. In another study, lower disease severity was associated with increased SARS-CoV-2-specific CD8+ and CD4+ T cells [32] T cell responses have been documented in humans following vaccination with either adenoviral vectors [30] or mRNA-based vaccines [31]. We show here that the SARS-CoV-2 MAPS vaccine elicited both IFN-γ secreting CD4^+^ and IFN-γ secreting CD8^+^ in NHP. While the optimal T cell profile remain to be determined, it is important to note that the vaccine studied here generated a balanced Th1, Th2 and Th17 CD4^+^ T cell response. Questions about durability and breadth of immune responses remain for approved mRNA vaccines, particularly given growing evidence to support a role of T cells in protection [8]. The cellular immune response generated in this study with the SARS-CoV-2 MAPS vaccine suggest that this vaccine can potentially demonstrated significant durability and further preclinical studies on the functional activity to the T cell responses will be performed in order to validate the advantages of this vaccine.

Furthermore, we demonstrate that SARS-CoV-2 MAPS vaccine was able to inhibit viral replication in both the upper and lower airways (BAL) by day 2 for most of the animals (5 out of 6), with no detectable virus in all animals by day 4. We observed a complete reduction in viral titers at day 2 for most of the animals (five out of six), with no detectable virus in all animals in the by day 4, comparable to what was observed in NHP challenge model with other vaccines using a challenge dose similar to the one used here [27,33].

Among the currently authorized COVID-19 vaccines, the two mRNA vaccines (BNT162b2 and mRNA-1273) have been the most widely used for both primary series and boosting. However, heterologous boosting, such as combining mRNA vaccines with adenovirus-vectored vaccines, has been shown to be advantageous in humans, leading to higher S-specific IgG, neutralizing antibodies, and S-specific CD4^+^ T cells than after homologous boosting [34,35,36]. Although mRNA vaccines are currently being used as boosters, other options, such as protein-based vaccines, could become more attractive, for tolerability and/or immunogenicity reasons.

In summary, we demonstrate here the potential of the MAPS platform to induce neutralizing antibody and T-cell responses directed against the S-RBD. As previously demonstrated in the context of *S. pneumonias* and *S. aureus* [9,12], the MAPS platform allows for the inclusion of multiple protein antigens in the same construct. This approach may be particularly attractive in the context of COVID-19 vaccines as incorporating multiple RBD variants could provide a powerful tool to combat emerging variants.

## Data Availability

Owing to privacy and ethical concerns, the datasets collected and/or analyzed during this work are not publicly available; however, anonymized data are available upon reasonable request from the corresponding author.
